# Theoretical and Experimental Investigation of Surface Topography Generation in Slow Tool Servo Ultra-Precision Machining of Freeform Surfaces

**DOI:** 10.3390/ma11122566

**Published:** 2018-12-17

**Authors:** Duo Li, Zheng Qiao, Karl Walton, Yutao Liu, Jiadai Xue, Bo Wang, Xiangqian Jiang

**Affiliations:** 1 Centre for Precision Engineering, Harbin Institute of Technology, Harbin 150006, China; qiaozhengyunlong@126.com (Z.Q.); 16B908095@stu.hit.edu.cn (Y.L.); brucexjd@hit.edu.cn (J.X.); 2 EPSRC Future Metrology Hub, University of Huddersfield, HD1 3DH Huddersfield, UK; K.Walton@hud.ac.uk (K.W.); x.jiang@hud.ac.uk (X.J.)

**Keywords:** ultra-precision machining, slow tool servo, surface topography, simulation, microlens array, sinusoidal grid

## Abstract

Freeform surfaces are featured with superior optical and physical properties and are widely adopted in advanced optical systems. Slow tool servo (STS) ultra-precision machining is an enabling manufacturing technology for fabrication of non-rotationally symmetric surfaces. This work presents a theoretical and experimental study of surface topography generation in STS machining of freeform surfaces. To achieve the nanometric surface topography, a systematic approach for tool path generation was investigated, including tool path planning, tool geometry selection, and tool radius compensation. The tool radius compensation is performed only in one direction to ensure no high frequency motion is imposed on the non-dynamic axis. The development of the surface generation simulation allows the prediction of the surface topography under various tool and machining variables. Furthermore, it provides an important means for better understanding the surface generation mechanism without the need for costly trial and error tests. Machining and measurement experiments of a sinusoidal grid and microlens array sample validated the proposed tool path generation and demonstrated the effectiveness of the STS machining process to fabricate freeform surfaces with nanometric topography. The measurement results also show a uniform topography distribution over the entire surface and agree well with the simulated results.

## 1. Introduction

Owing to the superior optical and physical properties, freeform surfaces are an important catalyst in the development of high-value-added photonics and telecommunication products, such as laser beam printers and scanners, head mounted displays, progressive lens molds, fiber optic connectors, and advanced automotive lighting systems [1,2,3,4]. Differing from conventional simple surfaces, freeform surfaces are more geometrically complex and normally have no symmetry in rotation or translation. To ensure the functionality of the freeform components, these surfaces are required to have sub-micrometer form accuracy and nanometer surface topography [5]. 

With the technical evolution in advanced manufacturing, ultra-precision machining technologies have been developed for the deterministic fabrication of high precision freeform surfaces including tool servo turning, ultra-precision raster milling, ultra-precision grinding and polishing [6,7,8,9]. Among them, slow tool servo (STS) machining provides an important means for generating optical freeform surfaces without the need for any subsequent processing. It has the advantages of simpler setup, faster cycle times and better machining accuracy over other techniques. Yi and Li [7] proposed an innovative diamond tool trajectory that allows the entire 5 × 5 microlens array to be fabricated in a single operation using STS machining. The machined microlens array surface was measured for both curve conformity and surface roughness and 34.5 nm Ra was achieved over 0.7 mm scanning length. Yin et al. [10] investigated the fabrication of off-axis aspheric surfaces using STS techniques. The form error of off-axis paraboloid caused by the tool centering error was analyzed in detail and verified by cutting experiment. RMS form error 0.063 μm of the overall region was achieved after adjusting the tool center. Mukaida and Yan [11] performed an experimental study of STS machining for single-crystal silicon microlens arrays. The surface error, material phase transformation, and cutting force characteristics were investigated experimentally. Spherical microlens arrays with a form error of 300 nm PV (peak to valley) and surface roughness of 6 nm Sa (arithmetical mean height of a surface) were successfully fabricated. Chen et al. [12] developed a model of three dimensional tool shape compensation for generating tool path in STS diamond turning of an asymmetrically toric surface for an astigmatic contact lens. The form accuracy of the freeform surface was evaluated by an ultra-high accuracy 3D profilometer. The form error was less than 0.5 μm both in the X and Y direction after the correction process.

Most studies have focused on the experimental investigation of STS machining of various types of freeform surfaces and the evaluation of form accuracy. However, surface topography should be studied [13,14], which is closely related to its functional performance, such as the diffractive properties of freeform optics [15,16]. Little systematic work has been reported about theoretical and experimental study of surface topography generation in STS machining of freeform surfaces. A successful STS machining depends largely on the selection of machining variables, tool parameters, and machining trajectories. Moreover, surface topography generation simulation offers a cost effective solution to select optimal cutting conditions, to predict the surface quality and to understand the machining phenomenon. A trial-and-error cutting approach is not economic because it is time consuming and costly [17].

This work presents a theoretical and experimental study of surface topography generation in STS machining of freeform surfaces. Several key machining aspects including tool path planning, selection of cutting tool geometries, and the tool radius compensation method, are discussed in detail. Moreover, surface generation simulation is proposed to theoretically investigate topography generation during the machining process. Finally, machining and measurement experiments of typical freeform surfaces are carried out to validate the effectiveness of the proposed methodology.

## 2. Tool Path Generation

**Figure 1:**
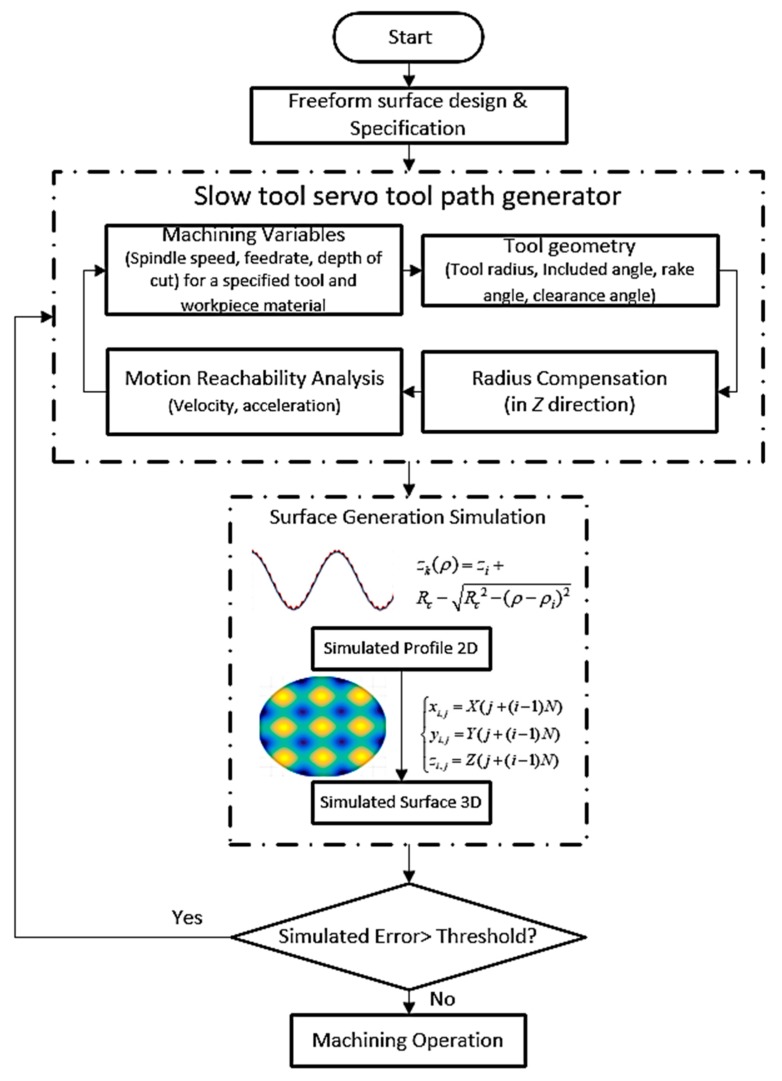
Workflow for slow tool servo (STS) tool path generation.

The workflow of STS machining of freeform surfaces is proposed as illustrated in Figure 1. According to the design and specification of freeform surfaces, the first task is to generate machining trajectories. In the initial stage, machining variables are selected to meet the targeted production requirement. Next, tool interference analysis is conducted to check if the diamond cutting tool can fully access the proposed machined features. To eliminate any overcutting phenomenon, tool radius compensation needs to be performed on the ideal tool path. Subsequently, the motion of machine tool axes is analyzed for its reachability of the modified tool path. In the second stage, numerical modelling is carried out which provides an important means to predict theoretical surface generation without the need for costly trial and error tests. Profile topography is generated by the intersection of a tool tip profile along the feeding direction and surface topography is formulated by a combination of all the radial sectional profiles along each angle. The simulated surface error is used as feedback information to guide the tool path generation processes. If it is less than the pre-defined value, the machining operation will be carried out.

### 2.1. STS Machining Principle

**Figure 2:**
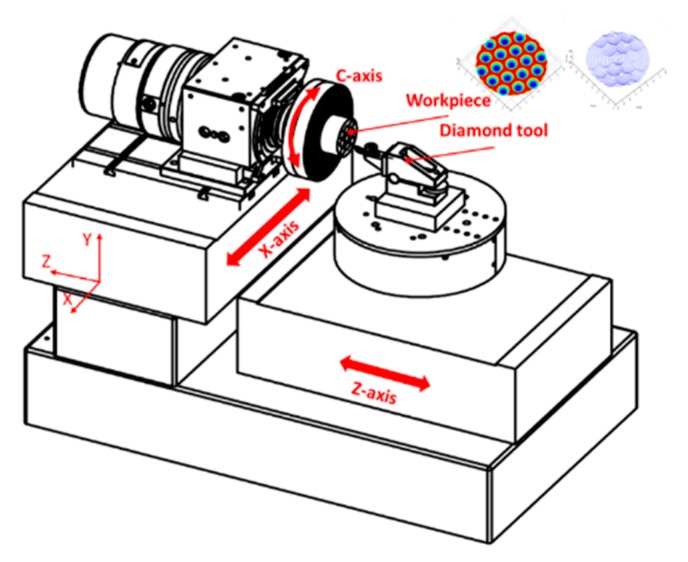
The schematic of the STS machining setup.

The conventional single point diamond turning (SPDT) process utilizes two linear axes for contouring motion with a velocity controlled spindle. Therefore, only rotational symmetric surfaces can be fabricated. As an adaption of conventional SPDT, the STS technique enables the spindle to actuate in a position controlled mode (also called C axis mode). The schematic of the STS machining setup is shown in Figure 2. An arbitrary 3D tool path for non-rotationally symmetric freeform surfaces can be achieved when the X axis, Z axis, and C axis move simultaneously following a given set of numerical motion commands. In most applications, the workpiece is mounted on the C axis while a diamond tool is installed on the Z axis, which needs to oscillate both forward and reverse in servo-synchronization with the angular position of the C axis and translational position of the X axis. 

**Figure 3:**
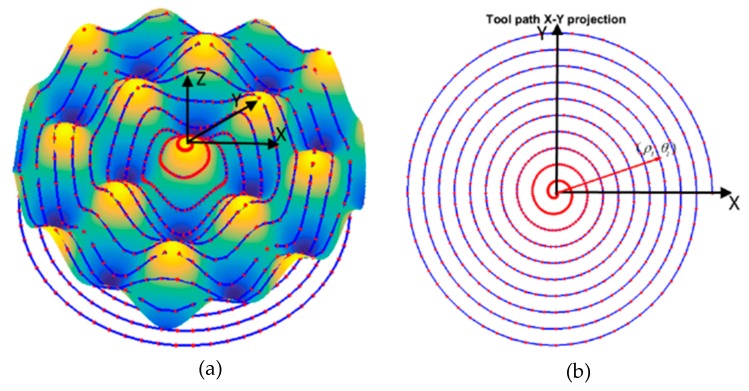
(**a**) STS ideal tool path and (**b**) X–Y projection.

In STS machining, the coordinate system is described as a cylindrical coordinate. The tool path projection on the X–Y plane is an equivalent spiral curve no matter how complex the surface is (as shown in Figure 3). The discrete points are equal-angle spaced for simple computation and control. 

In the X–Y plane, the spiral curve can be described mathematically as follows:

(1) $$\left\{ \begin{array}{l} \rho_{i}=R_{w}-(i-1)\frac{f}{S\cdot N_{\theta }}\\ \theta_{i}=(i-1)\frac{2\pi }{N_{\theta }}\\ i=1,2,\cdots ,\frac{R_{w}\cdot S\cdot N_{\theta }}{f}+1 \end{array}\right.$$

where *ρ_i_* is the radial distance (cylindrical coordinate) in mm, *θ_i_* is the polar angle (cylindrical coordinate) in radians, *R_w_* is the radius of workpiece in mm, *i* is the number of control points, *f* is the feedrate in mm/min, *S* is the C axis rotational speed in revolutions per minute (rpm), and *N_θ_* is the number of programmed points per revolution. However, the surface model to be fabricated is often expressed in a Cartesian coordinate (*x, y, z*) system. Under right-hand coordinate convention, the transformation between the two coordinate systems is as follows:

(2) $$\left\{ \begin{array}{l} x_{i}=\rho_{i}\cos(\theta_{i})\\ y_{i}=\rho_{i}\sin(\theta_{i})\\ z_{i}=F(x_{i},y_{i})=F(\rho_{i}\cos(\theta_{i}),\rho_{i}\sin(\theta_{i})) \end{array}\right.$$

where (*x_i_, y_i_, z_i_*) are the surface model points and *F(∙)* is the surface description. To illustrate the tool path generation principle, an STS ideal tool path (*ρ_i_*, *θ_i_*, *z_i_*) for a typical freeform surface (sinusoidal grid) can be generated. The surface is mathematically expressed as,

(3) $$z=A_{x}\cos(\frac{2\pi }{\lambda_{x}}x+\varphi_{x})+A_{y}\cos(\frac{2\pi }{\lambda_{y}}y+\varphi_{y})$$

where *A_x_* and *A_y_* are the amplitudes in the *X* and *Y* direction. *λ_x_* and *λ_y_* are the wavelength in the *X* and *Y* direction. *φ_x_* and *φ_y_* are the phase in the *X* and *Y* direction. Figure 3 shows the generated tool path and its spiral *X–Y* projection.

### 2.2. Tool Geometry Selection

**Figure 4:**
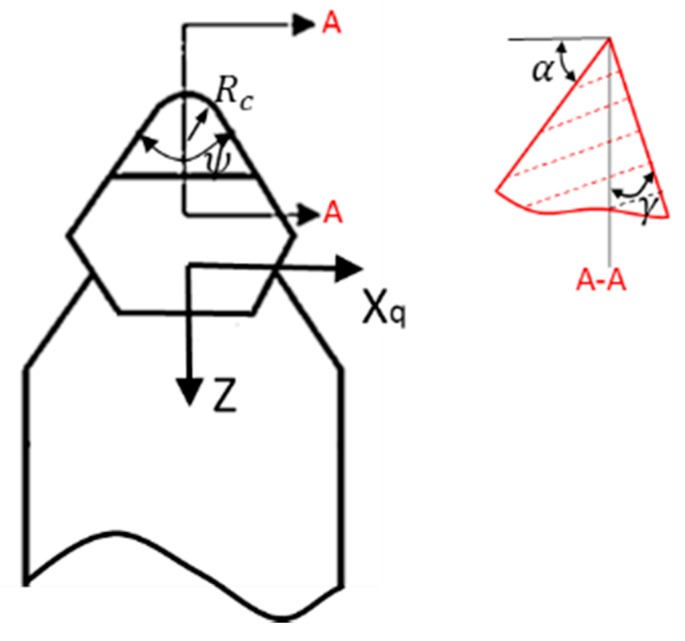
Geometric parameters of a typical diamond cutting tool.

Tool geometries should be carefully selected to guarantee the accessibility to the features of the proposed freeform surfaces. As shown in Figure 4, geometric parameters of a typical diamond cutting tool include the tool radius *R_c_*, the included angle *ψ*, the rake angle *γ*, and the clearance angle *α*.

**Figure 5:**
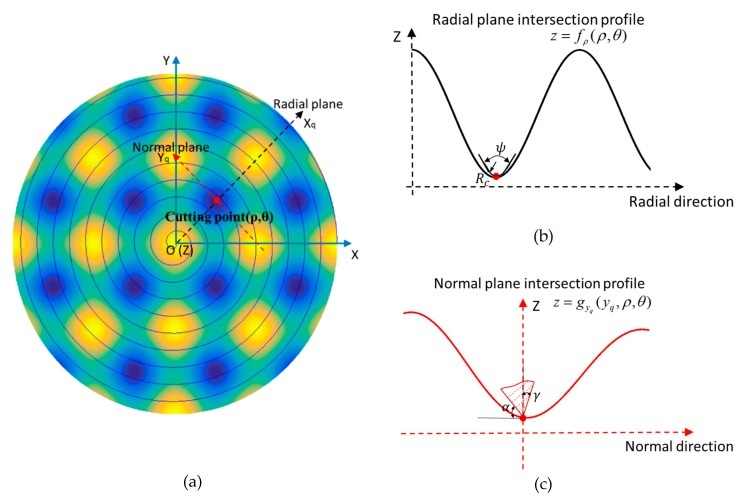
Schematic of tool geometry selection for STS freeform machining. (**a**) Radial and normal cutting plane; (**b**) Radial sectional profile; (**c**) Normal sectional profile.

The schematic for tool geometry selection in STS freeform machining is illustrated in Figure 5. For every cutting point (red dot in the plot), a radial cutting plane is determined by the Z axis and the cutting point while a normal plane is perpendicular to the radial plane and crosses the cutting point (shown in Figure 5a). Proper tool parameters should be selected to avoid interference with the machining surfaces in both planes. *R_c_* and *ψ* are calculated in the radial cutting plane, whereas *γ* and *α* are calculated in the normal plane.

As shown in Figure 5b, along each sectional profile $f_{\rho }\left(\rho ,\theta \right)$ in the radial plane, the tool tip radius *R_c_* should be small enough so that the tool is accessible to all the profile features while its critical value is determined by the minimum radius of curvature for all the cutting points. The included angle *ψ* should be large enough to make sure the cutting edge always stays in contact with the machining surface and its critical value is determined by the maximum value of the angle of inclination along the radial intersection profiles. The two conditions can be mathematically expressed as follows:

(4) $$\left\{ \begin{array}{ll} R_{c}\leq \min\left\{\frac{{\left(1+{\left(f_{\rho }^{\prime }(\rho ,\theta )\right)}^{2}\right)}^{\frac{3}{2}}}{f_{\rho }^{\prime \prime }(\rho ,\theta )}\right\} & 0<\rho \leq R\\ \psi \geq 2\max\left\{\arctan(f_{\rho }^{\prime }(\rho ,\theta ))\right\} & 0<\theta \leq 2\pi \end{array}\right.$$

where $f_{\rho }^{\prime }\left(\rho ,\theta \right)$ and $f_{\rho }^{\prime \prime }\left(\rho ,\theta \right)$ are respectively the first derivative and second order derivative of the radial intersectional profile $f_{\rho }\left(\rho ,\theta \right)$. To calculate the limit of the tool rake angle and the clearance angle, the intersection profile $g_{y_{q}}\left(y_{q},\rho ,\theta \right)$ in the normal plane is obtained in the normal plane perpendicular to the radial plane (the red curve shown in Figure 5c). The tool rake face and flank should not interfere the machined surface. Thus, the following conditions must be met:

(5) $$\left\{ \begin{array}{l} \gamma \geq \max\left\{\arctan(g_{y_{q}}^{\prime }(y_{q},\rho ,\theta ))-\frac{\pi }{2}\right\}\\ \alpha \geq \max\left\{-\arctan(g_{y_{q}}^{\prime }(y_{q},\rho ,\theta ))\right\} \end{array}\right.$$

where $g_{y_{q}}{}^{\prime }\left(y_{q},\rho ,\theta \right)$ is the first derivative of the normal plane intersectional profile. Besides the accessibility issue, the effect of the tool tip on the surface generation needs to be considered, which is discussed in the following section.

### 2.3. Tool Radius Compensation

**Figure 6:**
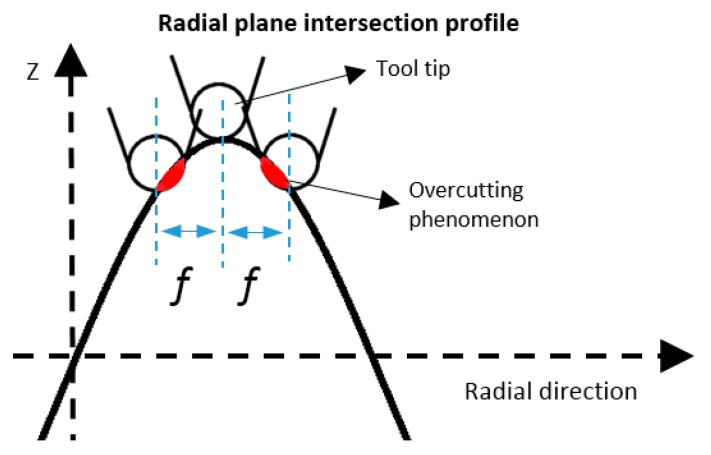
Schematic of overcutting phenomenon caused by circular tool tip.

Due to the circular geometry of the diamond tool tip, the cutting edge will cause overcut on the machined surface if the tool path is programmed based on the ideal infinitely sharp profile. Such an effect is illustrated in Figure 6. The red area shows the overcutting phenomenon, which would deteriorate the surface accuracy. 

**Figure 7:**
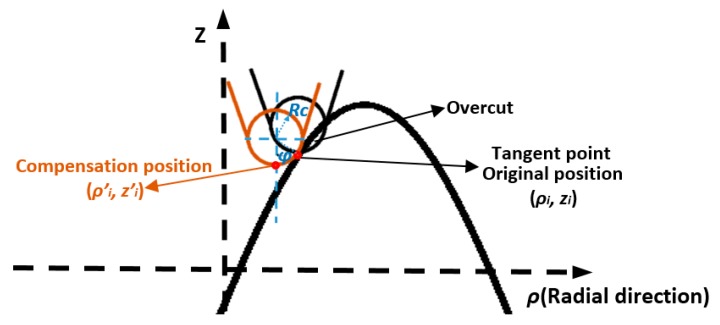
Tool radius compensation using normal direction method.

To avoid overcut on a machined surface, tool radius compensation is performed so that the circular tool edge should always be tangent to the intersection profile in each radial plane. Conventionally, tool radius compensation is performed in the normal direction on the cutting points [18,19]. The normal method is illustrated in Figure 7. The black tool tip shows the original programmed position. To compensate the overcut, the cutting position (red dot) is shifted so that the tool edge profile contacts tangential to the surface profile as indicated by the orange tool tip. The center of the circular tool edge is along the normal direction of the cutting point.

For a given radial intersection profile $f_{\rho }\left(\rho ,\theta \right)$, the relationship between the original cutting point $\left(\rho_{i},z_{i}\right)$ and the compensation position $\left(\rho_{i}^{\prime },z_{i}^{\prime }\right)$ can be expressed as follows:

(6) $$\left\{ \begin{array}{l} \rho_{i}{}^{\prime }=\rho_{i}-R_{c}\sin(\varphi_{i})\\ z_{i}{}^{\prime }=z_{i}+R_{c}\cos(\varphi_{i})-R_{c}\\ \tan(\varphi_{i})=\frac{df_{\rho }(\rho )}{d\rho }|{}_{\rho =\rho_{i}} \end{array}\right.$$

where $\varphi_{i}$ is the slope angle at $\left(\rho_{i},z_{i}\right)$ in the intersection profile. Calculation of the slope angle is required at every cutting point as the slope of freeform surfaces varies along the radial direction as well as at different angles.

**Figure 8:**
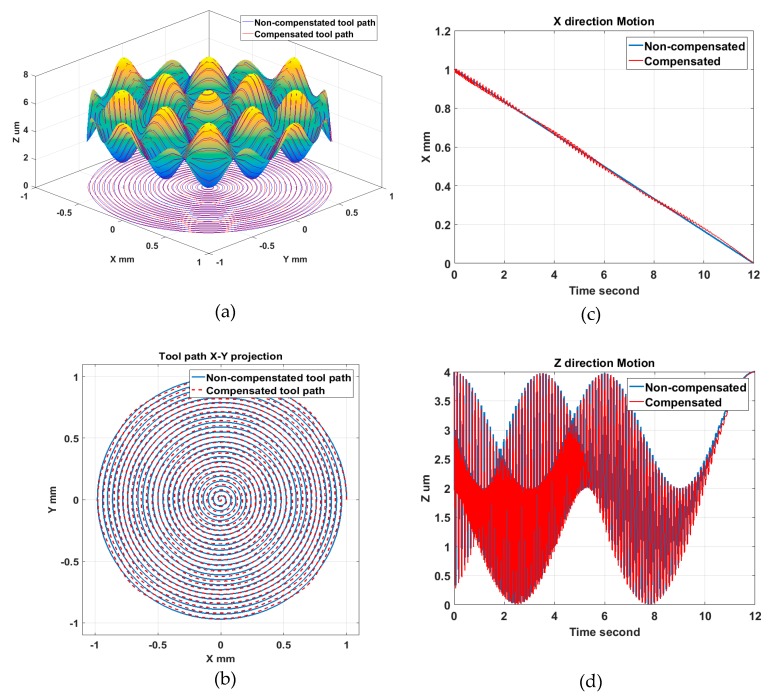
Normal direction compensation method and tool path analysis: (**a**) Tool path; (**b**) Tool path projection; (**c**) X axis motion; (**d**) Z axis motion.

To illustrate the tool radius compensation process, tool path generation was performed for a sinusoidal grid surface described by Equation (3). The surface design parameters were set to be *A_x_* = *A_y_* = 1 μm, *λ_x_* = *λ_y_* = 0.5 mm, *φ_x_* = *φ_y_* = 0. The machining variables were selected to be *f* = 5 mm/min, *S* = 100 rpm, *R_c_* = 0.5 mm. The compensated and uncompensated 3D tool path are shown in Figure 8a. Figure 8b indicates the X–Y projection of the compensated tool path and how it deviates from the original spiral trajectories. The motion analysis, illustrated in Figure 8c,d, shows additional motion components appearing on both the X and Z axis after the radius compensation in the normal direction. The disadvantage of the normal compensation method is that the tool tip shift is required to be performed in both the X and Z direction. Moreover, the shift value is not constant as the slope angle varies at different cutting points on freeform surfaces. The X feed will change along the radial direction using the normal compensation method. Therefore, high frequency motion of both the X and Z axis is required for tool radius compensation in the normal direction. For the configuration of the machine tool used in this work (shown in Figure 2), a heavy working spindle is mounted on the X axis and its dynamic response is thus limited.

**Figure 9:**
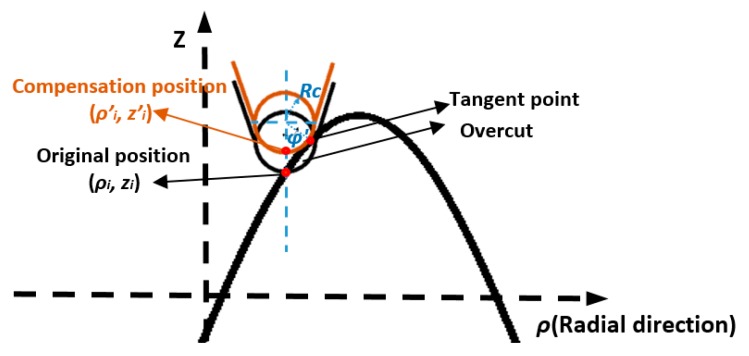
Tool radius compensation using the Z direction method.

Therefore, a modified tool radius compensation method is proposed as shown in Figure 9. In the Z direction compensation method, the tool tip only needs to shift along the Z direction until the cutting edge is tangential to the surface. The relationship between the original cutting point $\left(\rho_{i},z_{i}\right)$ and the compensation position $\left(\rho_{i}^{\prime },z_{i}^{\prime }\right)$ can be expressed as follows:

(7) $$\left\{ \begin{array}{l} \rho_{i}{}^{\prime }=\rho_{i}\\ z_{i}{}^{\prime }=z_{i}+\Delta z \end{array}\right.$$

(8) $$\{\begin{array}{c} \Delta z=\frac{Rc}{\cos\varphi^{\prime }}-Rc\\ \tan(\varphi^{\prime })=f_{{}_{\rho }}^{\prime }(\rho_{i}+R_{c}\sin(\varphi_{i})) \end{array}$$

where ΔZ is the tool shift value in the Z direction and $\varphi^{\prime }$ is the slope angle of the new tangential point. $\varphi^{\prime }$ is in an implicit equation and solved using Newton’s iterative algorithm [20]. 

**Figure 10:**
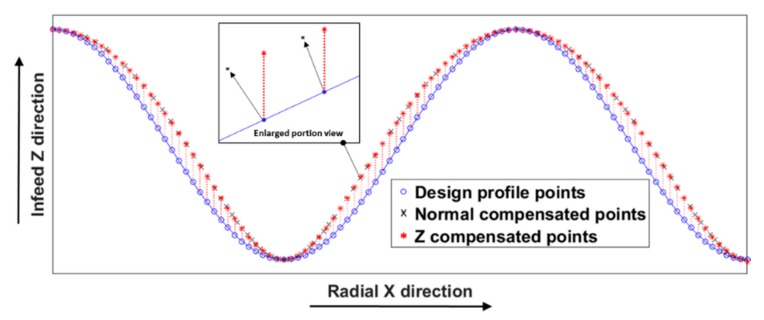
Comparison of two compensation methods along a radial profile.

The difference between the two compensation methods is simulated along a cosine radial profile and illustrated in Figure 10. The red dots show the tool position using the Z compensation method whereas the black dots represent the tool position using the normal compensation method. 

**Figure 11:**
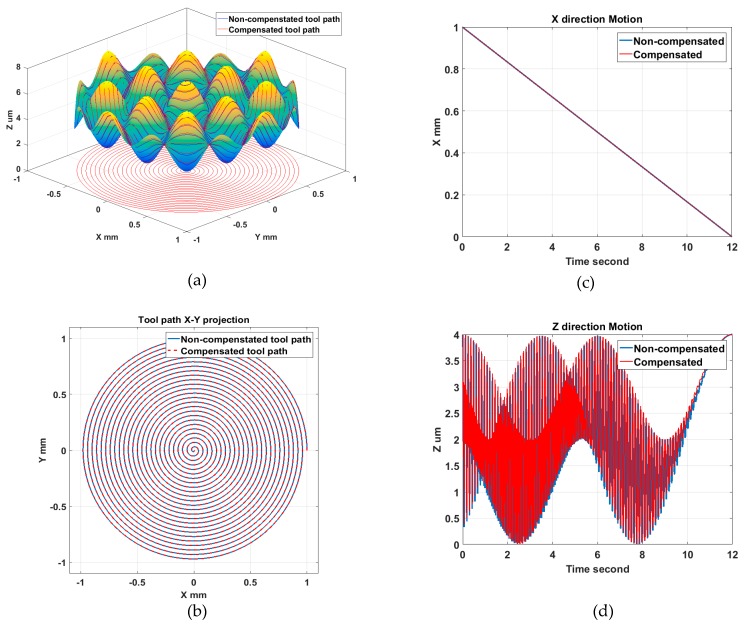
Z direction compensation method and tool path analysis: (**a**) Tool path; (**b**) Tool path projection; (**c**) X axis motion; (**d**) Z axis motion.

In addition, tool path simulation using the Z direction compensation method was carried out for the same sinusoidal grid surface presented above. As shown in Figure 11b, the X–Y projection of the compensated tool path coincides with the uncompensated one, which means the tool shift is only performed in the Z direction. The motion analysis in Figure 11c,d also validates that additional high frequency motion is avoided for the low-dynamic X axis. The X feed will not change along the radial direction using the Z compensation method. Therefore, the Z direction compensation method is considered stable and good for dynamic performance in STS machining of freeform surfaces.

## 3. Surface Generation Simulation and Analysis

### 3.1. Principles

**Figure 12:**
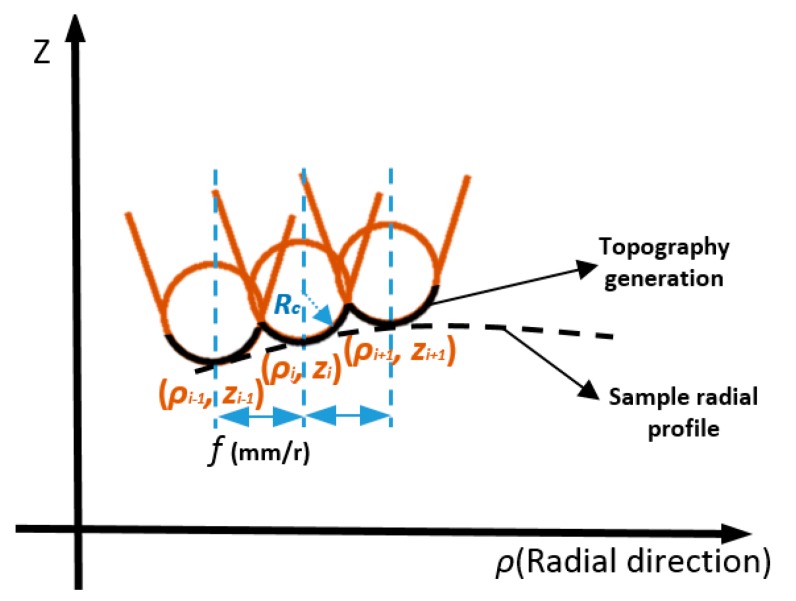
Schematic diagram of profile topography generation.

Surface generation simulation offers a cost effective solution to select optimal cutting conditions, to predict the surface quality and to understand the machining phenomenon without the need for costly trial and error machining tests. As illustrated in Figure 12, the successive tool positions are distributed at the interval of the feedrate along each radial intersection profile curve. Once the tool path is derived (the dashed line), the theoretical surface topography can be formed as the envelope of consecutive tool tip profiles along the feeding trajectory. Between the intersection points, the theoretical surface profile is a section of the circular tool tip profile (shown as black solid line).

Assuming the tool tip radius is *R_c_* and tool tip location is $\left(\rho_{i},z_{i}\right)$, the cutting profile can be expressed as:

(9) $$z(\rho )=z_{i}+R_{c}-\sqrt{R_{c}{}^{2}-{\left(\rho -\rho_{i}\right)}^{2}}$$

Thus, the profile topography height *h_envelope_* at radial position ρ can be calculated as the minimum value of all the cutting profiles:

(10) $$h_{envelope}(\rho )=\min\left\{z_{k}(\rho )\right\},\;k:i-1\;to\;i+1$$

**Figure 13:**
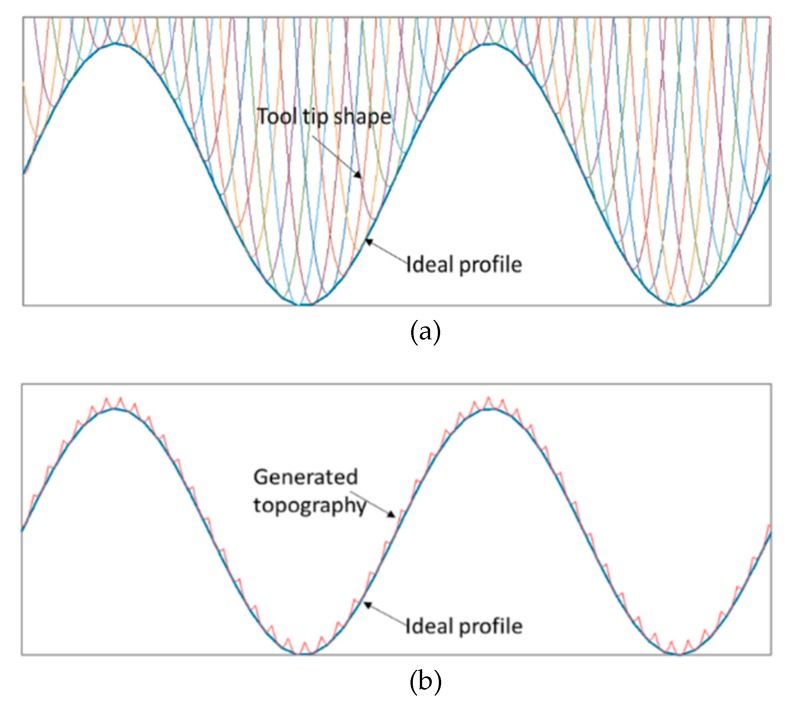
Simulation of topography generation along the radial profile: (**a**) Successive tool tip profiles along the feeding direction; (**b**) Resulting topography generation.

**Figure 14:**
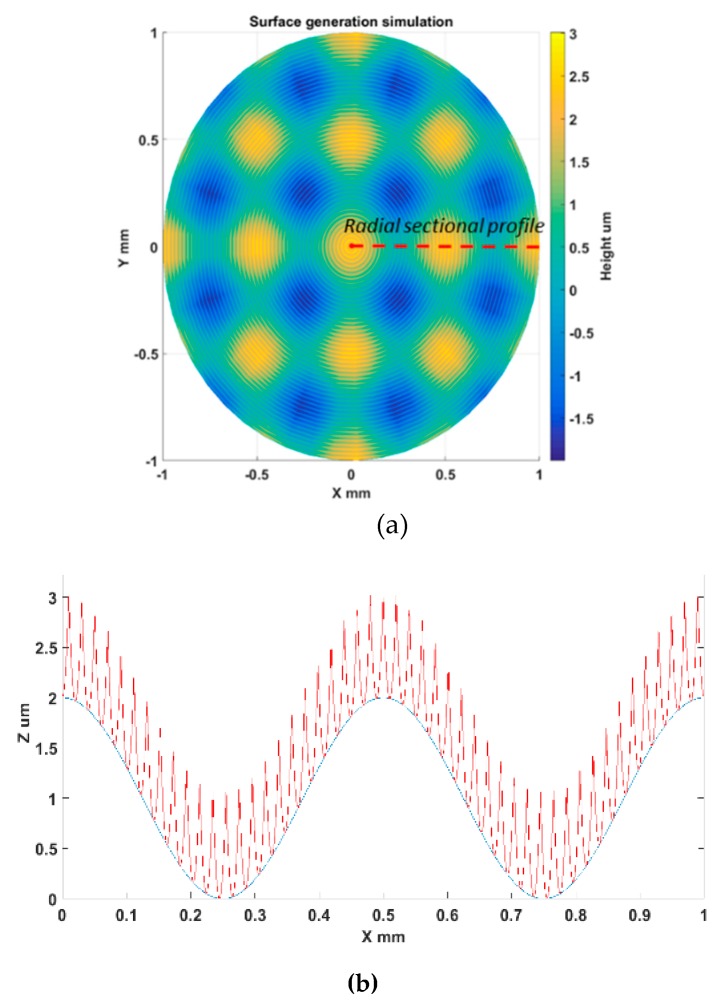
Simulation example of areal surface topography generation: (**a**) Surface generation simulation; (**b**) Radial section profile topography (0 degrees).

Taking a cosine radial profile as an example to validate the topography generation method, the result in Figure 13 clearly shows the successive tool tip profiles along the feeding direction and the resulting topography generation. The areal surface topography can be formulated by combination of all the radial intersection profile topography at each angle. With the above proposed method, generation simulation of the sinusoidal grid surface is performed. The surface parameters are the same as those in Section 2.3. For illustration purposes, the machining variables are selected to be *f* = 2 mm/min, *S* = 100 rpm, *R_c_* = 0.05 mm. Figure 14a,b respectively show the simulated areal surface topography and extracted profile topography at 0 degrees. The theoretical turning marks can be clearly seen on the simulated surface.

### 3.2. Simulation Analysis

With the established surface generation model, simulation analysis using MATLAB software is carried out in this section. The analysis is used to guide the selection of cutting parameters to achieve the targeted surface quality and better understand the machining processes as well. 

Without consideration of material effects, there are three processing parameters that influence the theoretical surface generation, which are tool radius *R_c_* (mm), feedrate *f* (mm/min), and spindle speed *S* (rpm). The relationship between processing parameters and surface quality is investigated with the aid of surface generation simulation. The investigation range is set as: *R_c_* 0.1–1 mm; *f* 0.2–1 mm/min; *S* 50–150 rpm. The root mean square height *S_q_* value (root mean square) is adopted to quantitatively describe the simulated surface quality.

**Figure 15:**
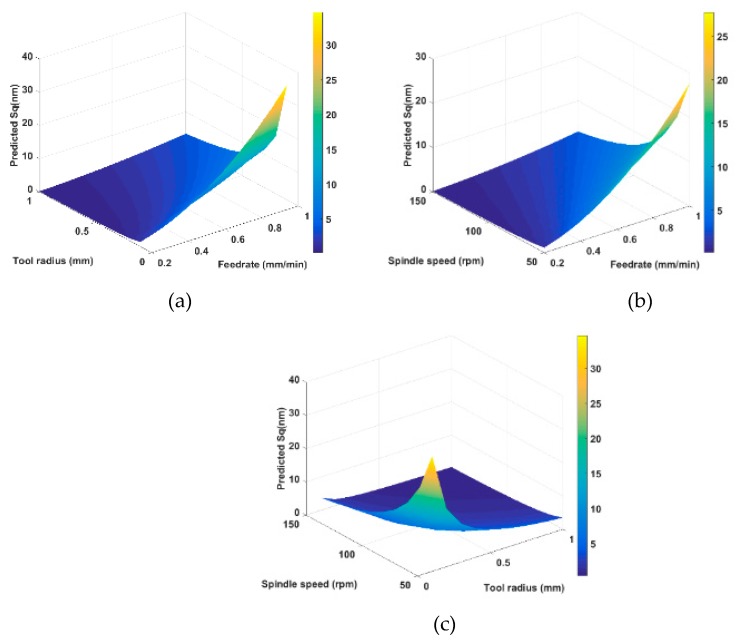
Relationship graphs between processing parameters and surface quality: (**a**) *R_c_* and *f* vs. *S_q_* (*S* = 100 rpm); (**b**) *S* and *f* vs. *S_q_* (*R_c_* = 0.5 mm); (**c**) *S* and *R_c_* vs. *S_q_* (*f* = 0.6 mm/min).

Figure 15a–c illustrates the quantitative relationship between processing parameters and surface quality using the surface generation simulation. From the simulation results, it can be concluded that a better surface finish (lower *S_q_* value) can be obtained under a higher spindle speed, a smaller federate, and a larger tool radius. In practice, it is better to choose a higher spindle speed rather than decreasing the feedrate. A lower feedrate would increase the machining time, decrease the tool life, and make the machining process vulnerable to environmental variations. However, higher spindle speed in STS machining requires a higher motion frequency and servo bandwidth, which is limited by the machine tool configuration and control strategy. The increase of tool radius results in the decrease of the *S_q_* value, the tool tip accessibility should be taken into consideration, which is discussed in Section 2.2. The relationship graphs are generated with the aid of surface generation simulation without costly trial and error experiments, which are useful to select optimized processing parameters to obtain a targeted surface quality.

Surface generation simulation also provides an important means for understanding the cutting phenomenon. In the following section, simulation analysis is performed to study the overcutting phenomenon and the effectiveness of tool radius compensation. 

**Figure 16:**
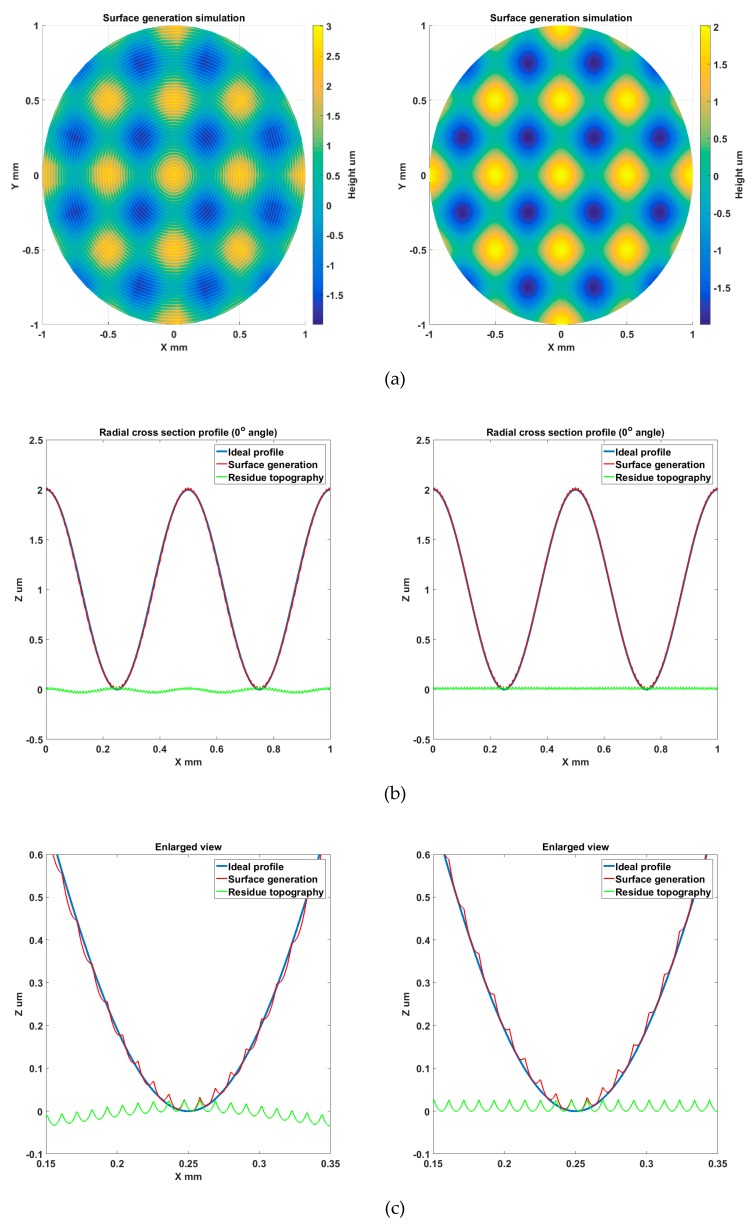

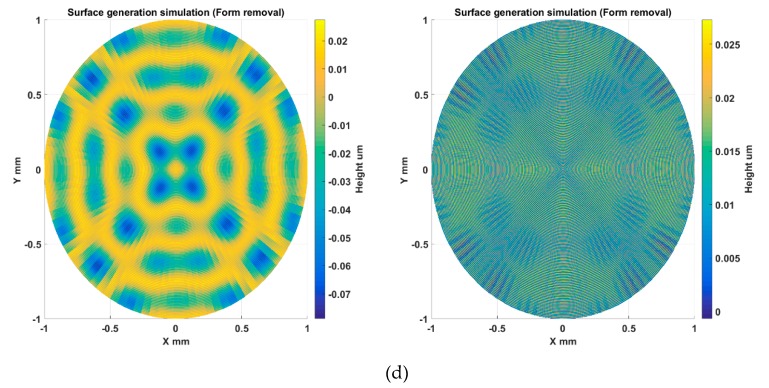
Simulation analysis of overcutting phenomenon and tool radius compensation (left column: without compensation, right column: with compensation): (**a**) Simulated surface generation; (**b**) Extracted radial profile topography; (**c**) Enlarged view of radial profile topography; (**d**) Simulated surface topography residual (after form removal).

The sinusoidal grid surface described by Equation (3) is used in the simulation. The surface design parameters are kept the same as those in Section 2.3. The machining variables are selected to be *f* = 5 mm/min, *S* = 50 rpm, *R_c_* = 0.5 mm. The simulation results are shown in Figure 16. Plots on the right and left show the simulation analysis of surface generation with and without tool radius compensation, respectively. As shown in the left plot of Figure 16b,c, the overcutting phenomenon can be clearly observed and waviness error components are induced on the machined surface due to tool tip overcut. In contrast, the overcutting phenomenon is avoided with the proposed tool radius compensation and waviness error components are eliminated, as shown in the right plot of Figure 16b–d which illustrates areal surface topography residual after form removal. The result also indicates that the pattern of induced waviness errors varies with the intersection angles. The study validated the proposed tool radius compensation method and effectiveness of simulation analysis to investigate the cutting phenomenon.

## 4. Experiments and Discussion

In order to show the effectiveness of the proposed STS machining methodology and surface topography generation, machining and measurement experiments of typical freeform surfaces (a sinusoidal grid and MLA surface) are carried out in the section.

### 4.1. Experimental Setup

**Figure 17:**
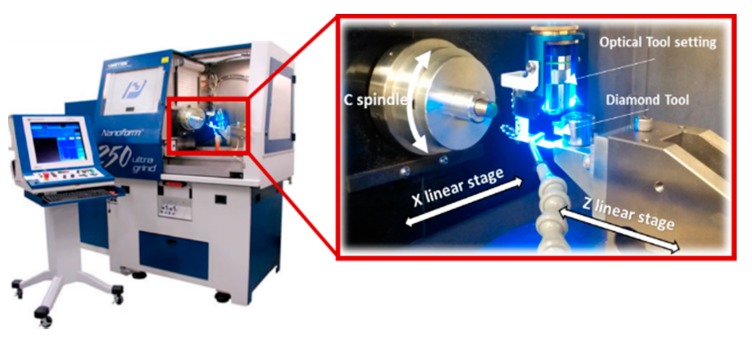
Experimental setup of STS machining.

**Figure 18:**
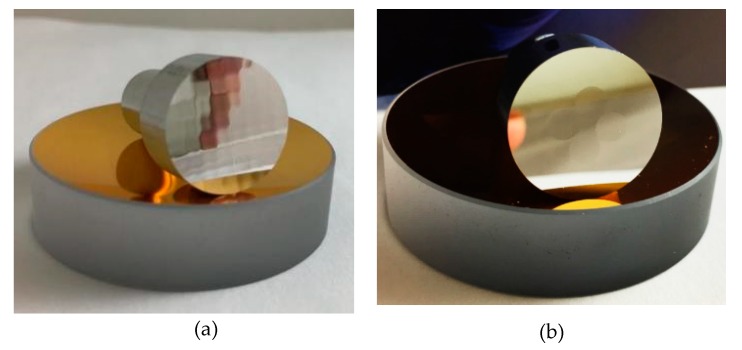
(**a**) Machined sinusoidal grid surface sample; (**b**) Machined micro-lens array (MLA) surface sample.

The machine tool used in the machining experiment is a Precitech Nanoform 250 Ultra Grind (Keene, NH, USA), which is shown in Figure 17. It can be used for diamond turning and ultra-precision grinding. The machine tool incorporates a finite element analysis (FEA) optimized dual frame for ultimate environmental isolation. A sealed natural granite base also provides excellent long term stability and vibration damping. Both the X and Z slides are equipped with hydrostatic oil bearing with symmetrical linear motor placement. The position of the X and Z axes is measured with linear laser scale encoders, which are capable of resolving 0.016 nm after signal subdivision. The straightness error for both the X and Z axes over the full travel is less than 0.2 μm. Under position controlled mode, maximum rotational speed of C axis can be 1500 rpm with a feedback resolution of 0.01 arc sec, while maintaining axial and radial error motion of less than 15 nm. The high precision and stability of the machine tool is a prerequisite for the ultra-precision machining process. The sample material used in the experiments is an aluminum alloy (Al6082) with a chemical composition of (0.7% Mn, 0.5% Fe, 0.9% Mg, 1% Si, 0.1% Cu, 0.1% Zn and 0.25% Cr). The material is of good machinability and its mechanical properties are listed in Table 1. The machined samples are shown in Figure 18.

### 4.2. Sinusoidal Grid Surface Machining 

**Figure 19:**
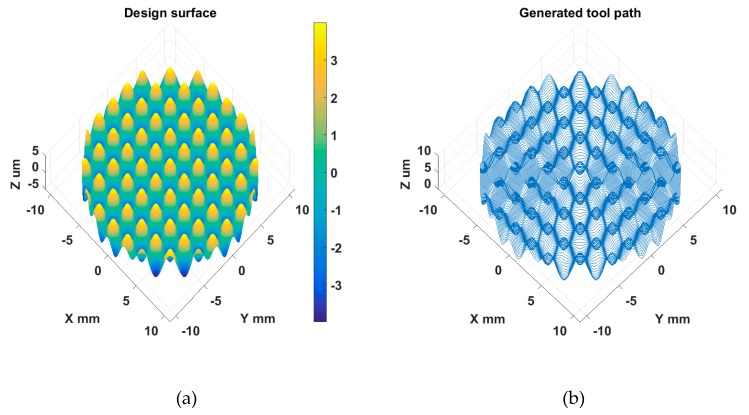
(**a**) Design and (**b**) STS tool path of sinusoidal grid surface.

A sinusoidal grid surface can be used for measurement of two-dimensional (2D) planar displacements [21]. The freeform surface is continuous and described mathematically by Equation (3). In the experiment, the design parameters were set to be *A_x_* = *A_y_* = 2 μm, *λ_x_* = *λ_y_* = 2.5 mm, *φ_x_* = *φ_y_* = 0. The machining and diamond cutting tool parameters are respectively listed in Table 2 and Table 3. With the analysis discussed in Section 2.2, the selected diamond tool can avoid interference with the machined surface. The proposed Z direction tool radius compensation was also performed on the ideal tool path to avoid the overcutting phenomenon. The design and STS tool path of the sinusoidal grid surface are illustrated in Figure 19a,b respectively. The sample was successfully machined, as shown in Figure 18a.

**Figure 20:**
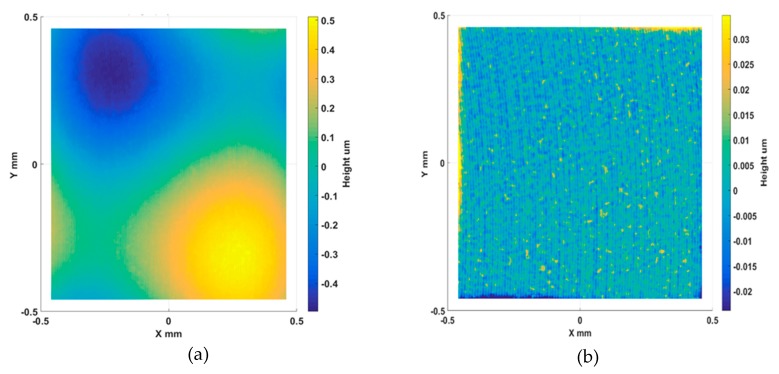
Machined sinusoidal grid surface CCI measurement: original (**a**); after form removal (**b**).

To inspect the machining quality, the sample was measured using a Talysurf Coherence Correlation Interferometry (CCI) 3000 (Taylor Hobson, Leicester, UK), equipped with a 20X microscope objective. The original and processed measurement results are shown in Figure 20a,b respectively. After filtering out the form component, the turning marks can be clearly observed from the CCI measurement. The surface topography was characterized by *S_q_*. 

**Figure 21:**
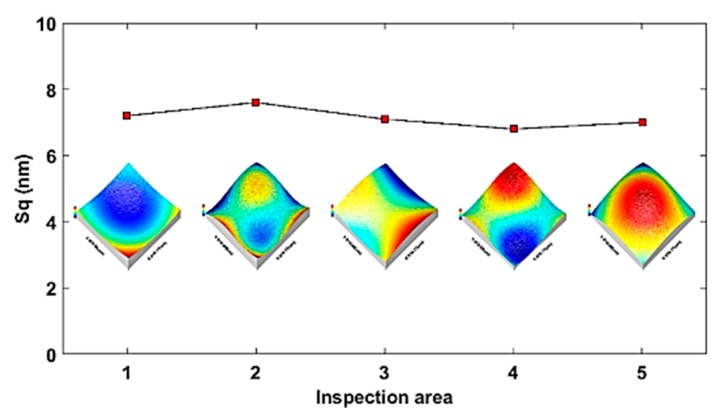
Topography distribution of sinusoid grid surface.

To examine the uniformity of the topography distribution, five areas were measured on the surfaces. The average *S_q_* is calculated as 7.1 nm and standard deviation is 0.30 nm. The measurement results (shown in Figure 21) indicate the machined topography of the continuous freeform surface is uniformly distributed over the surface and less than 10 nm, which meets the requirement for visible light optical application.

### 4.3. MLA Surface Machining

Micro-lens arrays (MLAs) play a key role in highly efficient light transmission [22]. The MLA surface is regarded as a type of structured freeform surface. It is composed of multiple elemental lenses, which are distributed in a specific pattern. In this experiment, the design parameters for MLA are listed in Table 4. 

**Figure 22:**
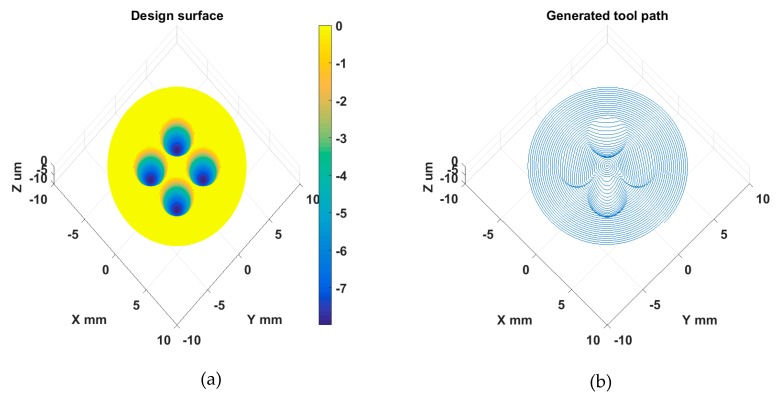
Design (**a**) and STS tool path (**b**) of MLA surface.

The same machining variables and tool used for the sinusoidal grid sample were used. The design and STS tool path of the MLA surface are respectively illustrated in Figure 22a,b. As shown in Figure 18b, the MLA sample was successfully machined to prove the effectiveness of STS machining of different types of freeform surfaces.

**Figure 23:**
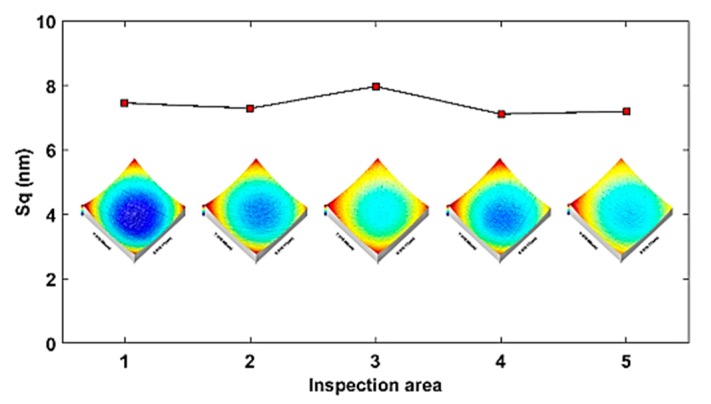
Topography distribution of MLA surface.

To inspect the machining quality, five areas in a different element lens were measured using Talysurf CCI 3000. The measurement results are shown in Figure 23. The average *S_q_* is calculated as 7.4 nm and the standard deviation is 0.34 nm. The measurement results indicate the machined topography of the structured freeform surface is also less than 10 nm and uniformly distributed.

The measured and simulated results of surface topography are also summarized in Table 5. The *S_q_* value of the actual measurement agrees with the simulated value, which proves the feasibility of the surface generation simulation.

## 5. Conclusions

This paper presents the investigation of surface topography generation in STS machining of freeform surfaces. Both theoretical and experimental investigation were conducted to prove the validity of the proposed tool path generation methodology and the effectiveness of surface generation simulation. The main conclusions can be drawn as follows:

(1) To avoid the overcut of a rounded tool tip, tool radius compensation in the Z direction was performed to ensure no high frequency motion is imposed on the dynamic-limited X axis. Tool path motion analysis validated the Z direction compensation method and it was shown to be advantageous over conventional normal direction compensation methods.
(2) The development of surface generation simulation allows prediction of the surface topography under various tool and machining variables. From the simulation results, it can be concluded that a better surface finish (lower *S_q_* value) can be obtained under a higher spindle speed, a smaller feedrate and a larger tool radius. The simulation analysis also reveals the surface generation mechanism (such as the overcutting phenomenon) without the need for costly trial and error tests. With the proposed tool radius compensation method, waviness error components resulting from the overcut can be totally eliminated.
(3) Machining experiments of a sinusoidal grid and MLA sample demonstrated the effectiveness of the proposed STS machining to fabricate optical freeform surfaces with a nanometric surface topography (less than 10 nm). The measurement results show uniform topography distribution over the entire surface and agree well with the simulation results.

The presented experimental results demonstrated the validity of the tool radius compensation method and surface generation simulation, which have the potential to be applied to other diamond machining processes (such as fly-cutting, grinding and fast tool servo) for freeform surfaces in order to improve the machining performance. These investigations will be of particular relevance to researchers in the ultra-precision freeform machining field.

## Figures and Tables

**Table 1:** Mechanical properties of the sample material (Al6082).

Parameters	Value
Density (g/cm3)	2.70
Modulus of elasticity (GPa)	70
Tensile strength (MPa)	260
Shear strength (MPa)	170
Thermal conductivity (W/m.K)	180

**Table 2:** Machining variables for sinusoidal grid surface.

Parameters	Value
Machining mode	STS
Spindle speed (rpm)	50
Feedrate (mm/min)	0.5
Cutting depth (μm)	3

**Table 3:** Diamond tool parameters.

Parameters	Value
Manufacturer	Contour fine tooling
Tool material	Single crystal
Tool tip radius (mm)	0.514
Rake angle (deg)	0
Clearance angle (deg)	10
Included angle (deg)	60

**Table 4:** Micro-lens array (MLA) design parameters.

Parameters	Value
Nominal feature shape	Sphere
Pattern	2 × 2
Center Spacing (mm)	4.243
Aperture radius (mm)	2
Chord height (μm)	8
Radius of curvature (mm)	250.004

**Table 5:** Surface topography *S_q_* by actual measurement and simulation.

Sample	Measured Average *S_q_* (nm)	Standard Deviation *S_q_* (nm)	Simulated *S_q_* (nm)
Sinusoidal grid	7.1	0.30	6.7
MLA	7.4	0.34	6.7
